# Contribution of submandibular gland and swallowing structure sparing to post-radiation therapy PEG dependence in oropharynx cancer patients treated with split-neck IMRT technique

**DOI:** 10.1186/s13014-016-0726-3

**Published:** 2016-11-15

**Authors:** Michael F. Gensheimer, Matthew Nyflot, George E. Laramore, Jay J. Liao, Upendra Parvathaneni

**Affiliations:** 1 Department of Radiation Oncology, Stanford University, Stanford, CA USA; 2 Department of Radiation Oncology, University of Washington Medical Center, 1959 NE Pacific St Box 356043, Seattle, WA 98195 USA

**Keywords:** Head and neck cancer, Oropharynx, Xerostomia, Dysphagia, Radiotherapy, Submandibular gland

## Abstract

**Background:**

Radiation therapy-related dysphagia is worsened by xerostomia. The submandibular glands (SMG) produce saliva rich in lubricating mucins, and sparing the SMG has been shown to reduce xerostomia. The goal of this study was to determine whether SMG sparing IMRT is associated with reduced post-treatment PEG dependence in locally advanced oropharynx cancer patients.

**Methods:**

Patients treated with definitive radiation therapy for oropharynx cancer were included in this retrospective study. Those with disease recurrence were excluded. Salivary glands and swallowing-related organs at risk, including pharyngeal constrictors, were contoured. Primary endpoint was time from end of radiation treatment to freedom from gastrostomy (PEG) tube dependence. Cox proportional hazards regression and logistic regression were used to assess influence of normal tissue doses on swallowing related endpoints.

**Results:**

Sixty-nine patients were included. All had stage III/IV disease and 97% received concurrent systemic therapy. Fifty-seven percent had contralateral SMG (cSMG) mean dose <50 Gy, a level shown to predict for xerostomia. Eighty four percent of patients had a PEG tube placed electively. On univariate analysis, the strongest predictor of time to freedom from PEG tube dependence was cSMG dose (HR 0.97 per Gy (95% CI 0.95–0.98), *p* < 0.0001). This relationship persisted on multivariate analysis (*p* = 0.052). The dose to superior and middle pharyngeal constrictor muscles, and larynx were also significant on univariate analysis. Patients with cSMG dose less than median (42 Gy, *n* = 34) had a significantly shorter time to freedom from PEG dependence: median 1.9 vs. 3.5 months, *p* < 0.0001. At 6 months, 3% of patients with cSMG dose < 42 Gy were PEG dependent compared to 31% with cSMG dose > 42 Gy (*p* = 0.002).

**Conclusions:**

Patients treated with cSMG sparing radiotherapy had significantly shorter time to PEG tube removal after treatment, suggesting a clinically meaningful reduction in subacute dysphagia compared to non-cSMG sparing treatment.

## Background

Dysphagia is a common late toxicity of radiation therapy for head and neck cancer and is detrimental to quality of life [1, 2]. A variety of risk factors for post-treatment dysphagia have been identified, including use of concurrent chemotherapy, advanced disease stage, and higher dose to pharyngeal constrictor muscles and other swallowing-related organs [3–5]. However, retrospective studies have shown little consistency in which individual swallowing-related structures are most important, and some studies failed to show a correlation between pharyngeal constrictor muscle dose and dysphagia when controlling for tumor location and other factors [1, 6, 7]. In a prospective study in oropharynx cancer patients, even when treatment was planned to minimize dose to swallowing structures (pharyngeal constrictors, larynx, and esophageal inlet), post-treatment dysphagia was common and persisted years after treatment [2]. Together, these data indicate a need for additional strategies to minimize post-treatment dysphagia.

Another common side effect of head and neck radiation therapy, xerostomia, exacerbates post-treatment dysphagia. Xerostomia is known to impede the normal swallowing process, especially for dry foods. In a study of post-radiation therapy patients with xerostomia, the swallowing steps of mastication and oral manipulation were compromised for solid foods [8]. Other studies have shown that post-treatment dysphagia and xerostomia are highly correlated, for both observer- and patient-reported measures [9–11]. Therefore, sparing the function of the salivary glands and reducing xerostomia could be a contributory strategy to reduce treatment-induced dysphagia. However, studies have not shown a consistent link between sparing the parotid gland alone and reducing dysphagia. In the PARSPORT randomized study comparing parotid sparing versus non-salivary sparing radiation therapy in head and neck cancer patients, late grade 3–4 dysphagia was actually more common in parotid spared patients at 12 months (9% vs. 5%) [12]. Sparing one or both submandibular glands (SMG) in addition to sparing the parotid glands has been shown to further reduce xerostomia in patients with oropharynx cancer [13, 14]. The SMGs produce saliva rich in mucins that act as a lubricant during the swallowing process [15]. While stimulated saliva is largely derived from the parotid, the optimal viscosity of the food bolus required for a successful swallow is also dependent on SMG derived mucinous saliva [15–18]. The goal of the present study was to determine whether contralateral submandibular gland (cSMG) sparing treatment is associated with decreased duration of PEG (percutaneous endoscopic gastrostomy) tube dependence, an objective endpoint that reflects subacute dysphagia.

## Methods

### Patient population

This was a retrospective study approved by the Institutional Review Board. Eligible patients were treated at our institution for oropharyngeal squamous cell carcinoma with definitive IMRT including bilateral neck treatment. Patients were required to have six months or longer follow-up in order to assess late toxicities. Those treated prior to late 2007 were not eligible because their computerized treatment plans were no longer accessible. Exclusion criteria included primary tumor resection prior to radiation therapy, any disease recurrence after radiation therapy, and prior radiation therapy to the head and neck region.

### Feeding tube placement

Per institutional policy, patients receiving concurrent chemoradiation had PEG tubes placed prophylactically prior to starting treatment unless they declined this procedure. For the patients who received radiation therapy without chemotherapy a case-by-case determination on prophylactic vs. as-needed (reactive) placement is made. After treatment, the feeding tube is removed once the patient is able to maintain his/her weight for 1–2 weeks using oral nutrition alone. These policies have not changed over the study period.

### Radiotherapy technique

The radiation therapy parameters used for these patients have been detailed previously [13]. In general, patients with primary tumor not encroaching on the cSMG and without contralateral nodal involvement had cSMG sparing treatment; in the other patients no attempt was made to reduce cSMG dose. When possible, bilateral parotid glands were spared to achieve mean dose < 24 Gy. In order to reduce larynx dose, a split field technique was used with a matched low-neck en face field with a larynx block. No specific attempt was made to spare the pharyngeal constrictor muscles, though there was an IMRT avoidance structure consisting of the oral cavity/oropharynx outside of the PTV.

A simultaneous integrated boost method was used. The highest risk target volume (PTV1) was generally prescribed 70 Gy in 33 fractions; the intermediate and low risk subclinical volumes (PTV2 and PTV3) received 62.7 and 57 Gy, respectively.

### Analysis

The primary endpoint was time from end of radiation treatment to freedom from PEG tube dependence. Patients who never required gastrostomy tube were scored as achieving the endpoint at time 0. The Kaplan-Meier method was used to estimate time to PEG tube removal. Patients were censored at time of last follow-up or death. Impact of clinical and dosimetric factors on this endpoint was assessed using univariate and multivariate Cox proportional hazards regression.

The secondary endpoint was presence/absence of grade ≥2 late (6 months or more after treatment completion) observer-rated dysphagia as defined by the Common Terminology Criteria for Adverse Events (CTCAE) version 4 [19]. Univariate and multivariate logistic regression was used to identify patient factors associated with the outcome. For multivariate analysis of both endpoints, only covariates with significant *p* values (*p* < 0.05) on univariate analysis were included due to sample size limitations.

Normal structures, including salivary glands and swallowing structures, were contoured for the study by one physician (M.G.) using established guidelines [20]. To improve the uniformity and efficiency of segmentation, atlas-based contouring was used in MIM version 6 (MIM Software, Cleveland, Ohio, USA). Contours were drawn on five patients’ CTs and propagated to the rest of the patients using deformable registration. Contours were then manually edited to ensure consistency and accuracy.

For the dosimetric analysis, we included the swallowing structures found to be significantly correlated with dysphagia in prior studies: the superior, middle, and inferior constrictor muscles, cricopharyngeal muscle, esophageal inlet muscles, and glottic/supraglottic larynx [1, 3, 5]. We also included the salivary structures: cSMG, parotid glands, and oral cavity (surrogate for minor salivary glands). For SMG dose, only the contralateral gland was used since the ipsilateral gland always overlapped the high dose planning target volume and would not be expected to have preserved function after treatment. For parotid gland dose, the mean of the two parotid gland doses was used since both parotid glands were usually spared and had similar doses. For all structures, only mean dose was used, because for both swallowing structures and salivary glands mean dose has been found to be highly predictive of patient outcomes [3, 21].

Differences in cSMG dose between patients with various clinical characteristics were assessed using the Wilcoxon rank-sum test. Statistical calculations were done with R software, version 3 (University of Auckland).

## Results

### Patients and treatments

Sixty-nine patients treated between Dec. 2007 and Feb. 2014 met the inclusion criteria. Patient characteristics are listed in Table 1. Almost all patients had stage IV disease (91%) and received concurrent systemic therapy (97%). The most common systemic therapy was high-dose cisplatin (*n* = 55).

**Table 1:** Patient characteristics

Characteristic	Number (%)
Age at treatment	
Mean	57
Range	25–73
Concurrent systemic therapy	
Yes	67 (97%)
No	2 (3%)
Disease subsite	
Tonsil	32 (46%)
Base of tongue	30 (43%)
Other/multiple	7 (10%)
AJCC stage	
III	6 (9%)
IVA	50 (72%)
IVB	13 (19%)
T stage	
1	5 (7%)
2	19 (28%)
3	17 (25%)
4a	21 (30%)
4b	7 (10%)
N stage	
0	2 (3%)
1	7 (10%)
2a	5 (7%)
2b	27 (39%)
2c	23 (33%)
3	5 (7%)

Dose to the primary tumor ranged from 66 to 72 Gy; 64 of 69 patients received a dose of 69.96 Gy in 33 fractions. Normal structure doses are listed in Table 2. In forty-three patients there was an IMRT objective for the cSMG. No patient had an IMRT objective for the ipsilateral SMG. Thirty-nine patients (57%) had cSMG mean dose < 50 Gy, a level which has been shown to predict preservation of gland function [21]. We examined whether baseline patient/tumor factors were associated with ability to perform cSMG sparing. Patients with bilateral nodal disease had higher cSMG doses than others (mean 59.3 vs. 36.7 Gy, *p* < 0.0001). Tumor stage (T1-3 vs. T4) and primary site (base of tongue vs. other) were not significantly associated with cSMG dose.

**Table 2:** Doses received by normal structures

Structure	Mean dose in Gy (range)
Contralateral submandibular gland	45.23 (18.16–74.24)
Parotid glands (bilateral)	32.50 (18.90–50.92)
Superior pharyngeal constrictor	61.05 (39.54–70.85)
Middle pharyngeal constrictor	62.74 (41.57–72.08)
Inferior pharyngeal constrictor	41.83 (21.90–70.04)
Esophageal inlet muscles	36.37 (10.23–62.91)
Cricopharyngeal muscle	30.85 (9.86–69.08)
Glottic/supraglottic larynx	46.70 (23.91–69.89)
Oral cavity	41.41 (17.37–72.00)

### Toxicity outcomes

Median follow-up was 27 months (range 7–78 months). Consistent with the inclusion criteria, no patient suffered disease recurrence. Two patients are deceased; cause of death was endocarditis in one and unknown in the other.

Fifty-eight patients (84%) had a gastrostomy tube placed prior to or during radiation therapy. Seventy-nine percent of these were placed before or during the first week of treatment, consistent with our department policy of prophylactic feeding tube placement for patients receiving chemoradiation. No patient had a feeding tube placed after completion of radiation therapy. In patients with a gastrostomy tube placed, median time to tube removal was 3.2 months. Gastrostomy tube dependence rate was 17% six months after treatment and 4% at twelve months. Two patients were tube dependent at last follow-up, 11 and 37 months after treatment.

**Fig. 1:**
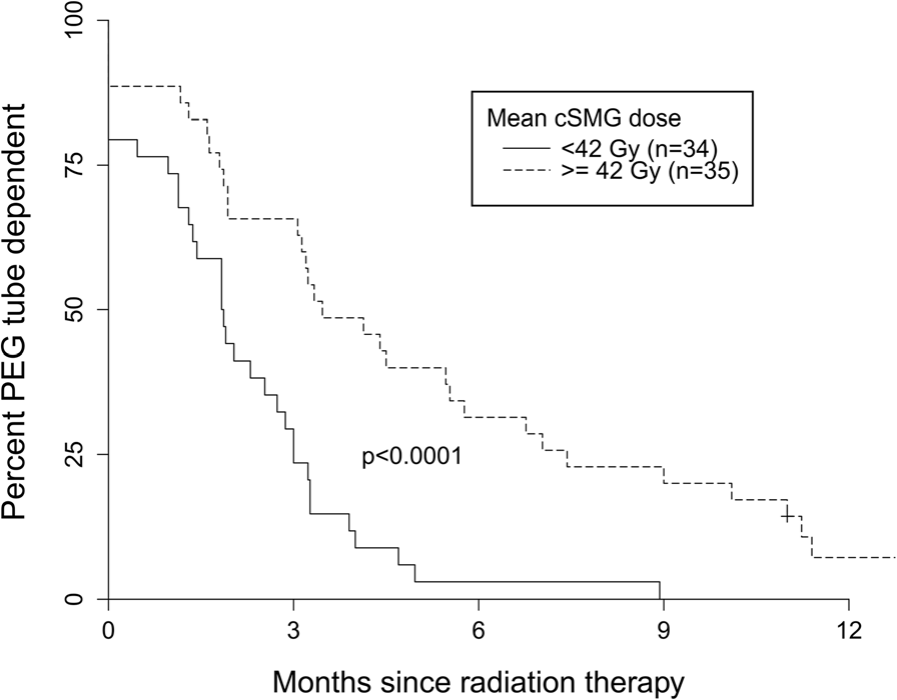
Freedom from PEG tube dependence by mean contralateral submandibular gland dose greater or less than median

Predictors of time to freedom from PEG dependence were examined using Cox proportional hazards regression (Table 3). On univariate analysis, the strongest predictor was cSMG dose (HR 0.97 per Gy, *p* < 0.0001). Other significant predictors were dose to the superior and middle pharyngeal constrictors, esophageal inlet muscles, larynx, and oral cavity. Parotid dose was not significant on any analysis. On multivariate analysis, cSMG dose was still the strongest predictor of this endpoint (HR 0.98 per Gy, *p* = 0.052). Median cSMG dose was 42.3 Gy. Patients with cSMG dose less than median (*n* = 34) had significantly shorter time to freedom from PEG tube dependence: median 1.9 vs. 3.5 months, *p* < 0.0001 by log-rank test (Fig. 1). At 6 months, only 3% (95% CI 0.4–20.2%) of patients with cSMG dose less than median were PEG tube dependent, versus 31% (95% CI 19.3–51.3%) of those with cSMG dose greater than median (*p* = 0.002). None of the patients with cSMG dose less than median were PEG tube dependent at 12 months.

**Table 3:** Predictors of time to freedom from PEG tube dependence (Cox proportional hazards model). Lower hazard ratio indicates later PEG tube removal

Variable (mean doses in Gy)	Univariate		Multivariate	
	Hazard ratio (95% CI)	*P* value	Hazard ratio (95% CI)	*P* value
Contralateral submandibular gland dose	0.97 (0.95–0.98)	**<0.0001**	0.98 (0.95–1.00)	0.052
Middle pharyngeal constrictor dose	0.92 (0.88–0.96)	**0.0002**	0.95 (0.89–1.02)	0.18
Superior pharyngeal constrictor dose	0.95 (0.92–0.98)	**0.003**	0.98 (0.93–1.03)	0.38
Glottic/supraglottic larynx dose	0.97 (0.94–0.99)	**0.007**	1.02 (0.98–1.07)	0.40
Esophageal inlet muscles dose	0.98 (0.97–1.00)	**0.03**	0.99 (0.97–1.01)	0.39
Oral cavity dose	0.97 (0.95–0.99)	**0.03**	1.00 (0.97–1.04)	0.86
Inferior pharyngeal constrictor dose	0.98 (0.96–1.00)	0.09		
T4 tumor stage	0.69 (0.42–1.15)	0.16		
Parotid glands mean dose	0.98 (0.94–1.02)	0.24		
Bilateral nodal involvement	0.77 (0.47–1.27)	0.31		
Cricopharyngeal muscle dose	0.99 (0.97–1.01)	0.34		
Age	1.00 (0.97–1.03)	0.96		

Statistically significant values are given in bold

The distribution of observer-rated late dysphagia (worst grade six months or later after treatment, CTCAE version 4) was: grade 0: 44 patients; grade 1: 10 patients; grade 2: 6 patients; grade 3: 9 patients. We examined predictors of late grade ≥2 dysphagia, defined as symptomatic and altered eating/swallowing (Table 4). On univariate analysis, cSMG dose was the strongest predictor of late dysphagia (OR 1.06 per Gy, *p* = 0.004). Other significant predictors were T4 vs. T1-3 tumor stage, bilateral nodal disease, superior constrictor dose, and oral cavity dose. On multivariate analysis, the only significant predictor of late dysphagia was T4 tumor stage (OR 4.60, *p* = 0.04).

**Table 4:** Predictors of grade ≥2 late observer-rated dysphagia (logistic regression)

Variable (mean doses in Gy)	Univariate		Multivariate	
	Odds ratio (95% CI)	*P* value	Odds ratio (95% CI)	*P* value
Contralateral submandibular gland dose	1.06 (1.02–1.10)	**0.004**	1.03 (0.98–1.09)	0.29
T4 tumor stage	5.99 (1.77–24.18)	**0.006**	4.60 (1.17–21.16)	**0.04**
Superior pharyngeal constrictor dose	1.27 (1.09–1.56)	**0.009**	1.15 (0.98–1.42)	0.12
Oral cavity dose	1.08 (1.01–1.16)	**0.03**	1.02 (0.94–1.12)	0.62
Bilateral nodal involvement	3.26 (1.02–11.18)	**0.05**	1.45 (0.28–7.56)	0.65
Glottic/supraglottic larynx dose	1.06 (1.00–1.12)	0.06		
Parotid glands mean dose	1.06 (0.99–1.15)	0.11		
Inferior pharyngeal constrictor dose	1.04 (0.99–1.09)	0.13		
Middle pharyngeal constrictor dose	1.08 (0.98–1.21)	0.15		
Cricopharyngeal muscle dose	1.02 (0.98–1.07)	0.35		
Age	1.02 (0.95–1.10)	0.56		
Esophageal inlet muscles dose	1.01 (0.97–1.05)	0.63		

Statistically significant values are given in bold

Thirty-nine patients (57%) had grade ≥2 observer-rated late xerostomia. Consistent with our prior report, patients with higher cSMG dose were more prone to grade ≥2 late xerostomia (OR 1.05 per Gy, *p* = 0.002) [13]. Oral cavity dose was also associated with late xerostomia (OR 1.05 per Gy, *p* = 0.05), but parotid gland dose was not (*p* = 0.51). Observer-rated late dysphagia and xerostomia were modestly correlated, with a phi coefficient of 0.32, *p* = 0.02 by the chi-square test.

## Discussion

We examined the influence of clinical and dosimetric factors on post-treatment PEG dependence in patients with locally advanced oropharynx cancer. cSMG dose was the strongest predictor of time to freedom from PEG tube dependence on both univariate and multivariate analysis. Patients with cSMG dose less than median (42 Gy) had a 3% rate of PEG tube dependence at 6 months and 0% at 12 months. To our knowledge, this is the first report of an objectively measured dysphagia related outcome, i.e., post treatment PEG dependence, after cSMG sparing bilateral neck IMRT.

Most studies correlating radiation dose to pharyngeal constrictor muscles and other swallowing-related organs (larynx, and esophageal inlet) did not include the salivary glands in their models of dysphagia [3, 6, 7]. During the process of swallowing a food bolus, in addition to the neuro-muscular structures involved, an optimal volume and viscosity of saliva, and adequate lubrication are necessary [8, 15–18]. While parotid derived stimulated saliva contributes to the volume of saliva, SMG derived saliva rich in mucins provides the viscosity and lubrication needed for swallowing of foods such as dry solids [15]. A prospective study of 93 patients treated with radiation therapy showed that post-treatment patient-reported xerostomia grade was a strong predictor of dysphagia grade [9]. Thus, it is plausible that minimizing xerostomia, by preserving the function of the salivary glands in addition to sparing the muscular swallowing structures, could reduce post-treatment dysphagia and PEG dependence.

Our analysis suggests that sparing the cSMG could be an important factor in minimizing detriment to the swallowing process. This is also supported by a study of 354 patients with mostly oropharynx and larynx primaries showing that SMG doses were significant predictors of post-treatment swallowing dysfunction on univariate analysis [5]. While parotid glands were routinely spared (in addition to cSMG) in our cohort, sparing the parotid glands alone is unlikely to improve swallowing function as parotid saliva is watery and lacks mucins [12].

The two endpoints in this study (freedom from gastrostomy tube dependence after treatment, and late observer-rated late dysphagia) have both been commonly used as indicators of dysphagia after radiation therapy [2, 4, 22]. The results for the two endpoints differed somewhat. While cSMG dose was predictive of both endpoints on univariate analysis, on multivariate analysis, cSMG dose was a strong predictor of the objective gastrostomy tube endpoint but not observer-rated dysphagia (*p* = 0.052, 0.29, respectively). The feeding tube endpoint may be more relevant to patients, for a few reasons. First, duration of feeding tube requirement correlates with reduced quality of life related to late dysphagia. In a study of head and neck cancer patients one year after treatment, having a feeding tube present at one year was associated with reduced quality of life in nine of twelve subscales [23]. Our patients’ 4% PEG tube dependence rate at 12 months is similar to the 1% rate reported in the constrictor sparing trial [2] and less than the 9% rate reported among stage III-IV patients receiving chemoradiation in a multi-institutional study [4].

Second, observer-rated dysphagia is a subjective endpoint and is known to correlate poorly with patient-reported dysphagia [24]. For instance, in a recent prospective study only 1% of patients had grade 2 or worse observer-scored dysphagia 24 months after radiation therapy, compared to over 50% of patients reporting dysphagia at the same time point [9].

**Fig. 2:**
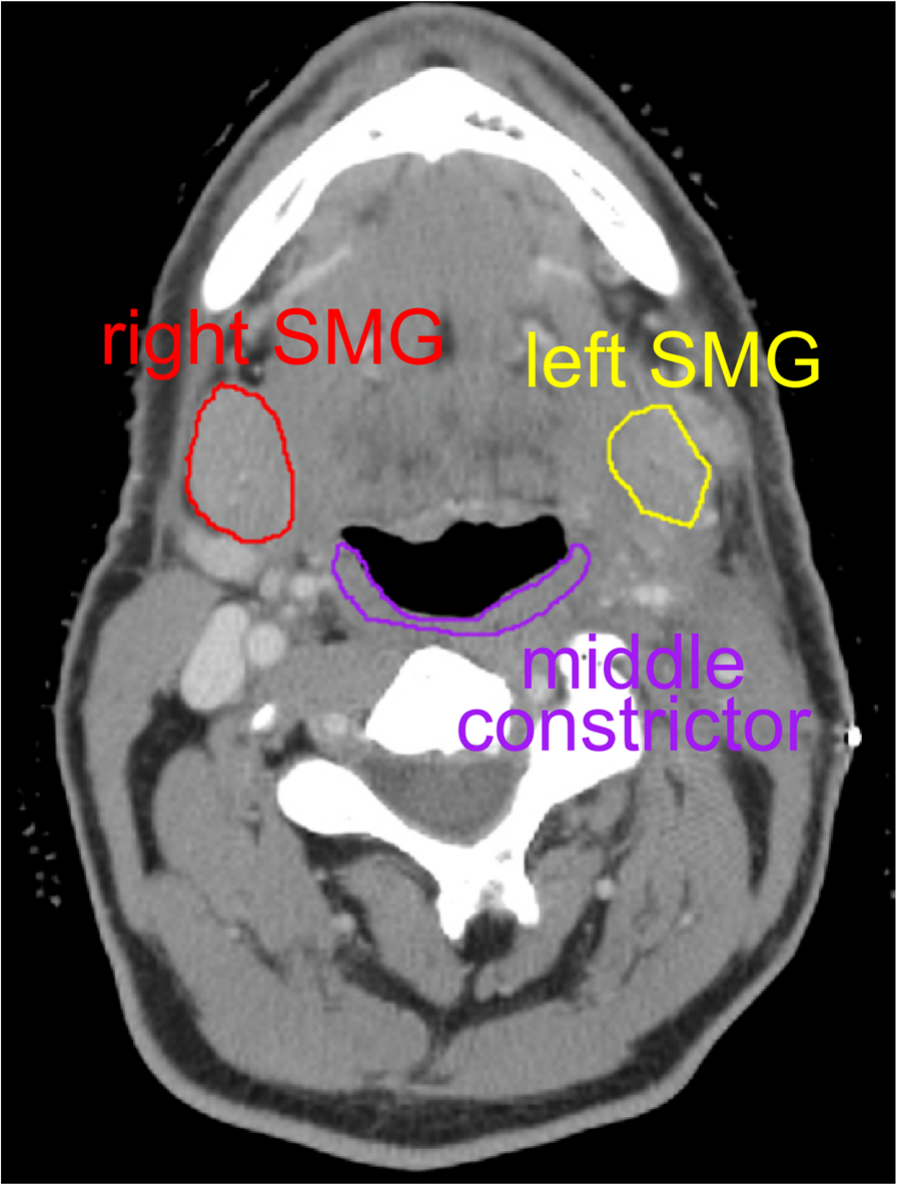
Relationship of submandibular glands to middle pharyngeal constrictor muscle. There may be a tradeoff in radiation treatment planning between aggressively sparing the constrictor muscles and the contralateral submandibular gland

An interesting issue could be the tradeoff between cSMG sparing and pharyngeal constrictor sparing. The submandibular glands are located at a similar craniocaudal location as the middle pharyngeal constrictor muscle (Fig. 2). A potential concern is that by aggressively sparing the cSMG instead of prioritizing pharyngeal constrictor sparing, the dose to swallowing muscles could increase, worsening dysphagia. A crude comparison shows that our patients’ swallowing structure doses are similar to those in a trial of constrictor muscle sparing IMRT in which the mean constrictor dose was 58 Gy, the mean larynx dose was 48 Gy, and the mean esophageal inlet dose was 34 Gy [2]. This suggests that cSMG sparing in our series did not overly increase dose to the swallowing structures, as we routinely spared the uninvolved oral cavity and oropharynx.

The main limitation of this study is the non-randomized nature of the cSMG sparing treatment, raising the possibility of bias and unmeasured confounders. Patients without bilateral nodal involvement were more likely to have cSMG sparing; nodal stage has been shown in some other series to be a predictor of post-treatment dysphagia [4]. In the present study, bilateral nodal disease was not a significant predictor of gastrostomy tube dependence and was not included in the multivariate regression. The lack of patient-reported quality of life outcomes is another limitation.

## Conclusions

This hypothesis-generating study shows that cSMG sparing radiotherapy may reduce post-treatment PEG dependence and dysphagia. We are currently conducting a prospective quality of life study to learn whether cSMG sparing is associated with improvement in patient-reported outcomes.

## Abbreviations

cSMG: contralateral submandibular gland
CT: Computed tomography
CTCAE: Common Terminology Criteria for Adverse Events
IMRT: Intensity-modulated radiation therapy
PEG: Percutaneous endoscopic gastrostomy
PTV: Planning target volume
SMG: Submandibular gland
